# Ankle arthritis predicts worse outcome in children with juvenile idiopathic arthritis

**DOI:** 10.1186/1546-0096-12-S1-P33

**Published:** 2014-09-17

**Authors:** Anna-Clara Esbjörnsson, Kristiina Aalto, Eva W Broström, Anders Fasth, Troels Herlin, Susan Nielsen, Ellen Nordal, Marite Rygg, Marek Zak, Lillemor Berntson

**Affiliations:** 1 Department of Women´s and Children´s Health, The Karolinska Institute, Stockholm, Sweden; 2 Department of Paediatrics, Children’s Hospital, Helsinki University Hospital, Helsinki, Finland; 3 Department of Paediatrics, University of Gothenburg, Gothenburg, Sweden; 4 Department of Paediatrics, Århus University Hospital, Århus, Denmark; 5 Pediatric Rheumatology Department, Copenhagen University Hospital, Rigshospitalet, Copenhagen, Denmark; 6 Department of Paediatrics, University Hospital of North Norway, Tromsø, Norway; 7 Department of Laboratory Medicine, Children’s and Women’s Health, and Department of Pediatrics , Norwegian University of Science and Technology, St.Olav’s Hospital, Trondheim, Norway; 8 Department of Paediatrics, Uppsala University Hospital, Uppsala, Sweden

## Introduction

The ankle joint is commonly involved in children with Juvenile Idiopathic Arthritis (JIA) and ankle arthritis predicts a more severe disease according to earlier studies. These studies have mainly been cross-sectional and the results are problematic to generalize to broader populations.

## Objectives

To evaluate the presence of ankle arthritis in children with JIA in a population-based cohort, to describe clinical characteristic in children with ankle arthritis and to evaluate the relation between ankle arthritis and remission status eight years after disease onset.

## Methods

In total 440 children with JIA were included prospectively in a population based cohort study. Data on remission was available for 427 of these children. The presence of ankle arthritis during an eight years follow-up period was analyzed in relation to remission data and clinical characteristics. Remission was defined according to the preliminary criteria by Wallace et al. 2004.

## Results

Of the 440 children with JIA, 251 (57%) experienced ankle arthritis during the first eight years of disease. Ankle arthritis was least common in the persistent oligoarticular category (25%) and the ankle joint was most commonly affected in children with the extended oligoarticular (83%) and polyarticular RF negative (85%) JIA. Children who developed ankle arthritis, were younger at disease onset (median age 4.5 (IQR 2.0-8.7) vs. 7.3 (IQR 3.5-10.8), p<0.001) and had more cumulative involved joints (median involved joints 10 (IQR 5-16) vs 2 (IQR 1-5), p<0.001), compared to those without ankle arthritis. Hind, mid and forefoot involvement were significantly more common in children with ankle involvement as compared to those without. The OR for not being in remission eight years after disease onset was 2.6 (95% CI:1.7-3.8, p<0.001) if the ankle joint was involved. After adjusting for other joints in the lower extremity the OR for not being in remission was 1.6 (95% CI: 1.1-2.5, p=0.03).

## Conclusion

- Ankle joint arthritis in children with JIA was associated with a young age of onset

- The ankle joint was frequently involved except for the persistent oligoarticular category

- The presence of ankle arthritis was related to failure to achieve remission

## Disclosure of interest

None declared.

